# Saponin treatment for eukaryotic DNA depletion alters the microbial DNA profiles by reducing the abundance of Gram-negative bacteria in metagenomics analyses

**DOI:** 10.20517/mrr.2023.02

**Published:** 2023-11-15

**Authors:** Giulia Longhi, Chiara Argentini, Federico Fontana, Chiara Tarracchini, Leonardo Mancabelli, Gabriele Andrea Lugli, Giulia Alessandri, Edith Lahner, Giulia Pivetta, Francesca Turroni, Marco Ventura, Christian Milani

**Affiliations:** ^1^Laboratory of Probiogenomics, Department of Chemistry, Life Sciences, and Environmental Sustainability, University of Parma, Parma 43124, Italy.; ^2^GenProbio Srl, Parma 43124, Italy.; ^3^Department of Medicine and Surgery, University of Parma, Parma 43124, Italy.; ^4^Microbiome Research Hub, University of Parma, Parma 43124, Italy.; ^5^Medical-Surgical Department of Clinical Sciences and Translational Medicine, Sant’Andrea Hospital, School of Medicine, University Sapienza, Rome 00185, Italy.

**Keywords:** Host DNA depletion, saponin, bacterial DNA enrichment, microbiome profiling

## Abstract

**Background:** Recent advances in microbiome sequencing techniques have provided new insights into the role of the microbiome on human health with potential diagnostic implications. However, these developments are often hampered by the presence of a large amount of human DNA interfering with the analysis of the bacterial content. Nowadays, extensive scientific literature focuses on eukaryotic DNA depletion methods, which successfully remove host DNA in microbiome studies, even if a precise assessment of the impact on bacterial DNA is often missing.

**Methods:** Here, we have investigated a saponin-based DNA isolation protocol commonly applied to different biological matrices to deplete the released host DNA.

**Results:** The bacterial DNA obtained was used to assess the relative abundance of bacterial and human DNA, revealing that the inclusion of 2.5% wt/vol saponin allowed the depletion of most of the host’s DNA in favor of bacterial DNA enrichment. However, shotgun metagenomic sequencing showed inaccurate microbial profiles of the DNA samples, highlighting an erroneous increase in Gram-positive DNA. Even the application of 0.0125% wt/vol saponin altered the bacterial profile by depleting Gram-negative bacteria, resulting in an overall increase of Gram-positive bacterial DNA.

**Conclusion:** The application of the saponin-based protocol drastically changes the detection of the microbial composition of human-related biological specimens. In this context, we revealed that saponin targets not only host cells but also specific bacterial cells, thus inducing a drastic reduction in the profiling of Gram-negative bacterial DNA.

## INTRODUCTION

Microbiome research, especially the detection of microorganisms by molecular techniques, has become a fundamental tool for investigating host-associated bacteria, such as those harbored by veterinary or human clinical samples^[1,2]^. Next-generation sequencing (NGS) approaches now enable the identification of slow-growing, non-cultivable, or non-viable bacteria contained in clinical specimens without relying on conventional culture-based identification methods based on the isolation of microorganisms by *in vitro* growth^[3,4]^. Basic PCR amplicon systems were exploited for DNA sequencing cost reasons, amplifying a specific gene for genus-level phylogenetic profiling purposes^[5]^. The study of microbial composition is rapidly progressing due to recent advances in DNA sequencing platforms employed for shotgun metagenomics sequencing approaches, and concomitantly lowering of the costs of DNA sequencing, thus guaranteeing the investigation of species-level taxonomic profiles along with genetic and functional information of host-associated microbiomes^[6]^.

However, despite using protocols specific for bacterial DNA extraction, the huge amount of host DNA in many biological samples analyzed generates no reliable metagenomic data where the considerable amount of eukaryotic DNA hinders the microbial sequences. High human-microbial DNA ratios have been reported for skin swabs as well as sputum^[7]^, saliva^[8]^, oral swabs^[9]^, vaginal samples^[10]^, and human biopsies^[11]^, making it difficult to investigate the resident microbial population. The human/microbial DNA ratio can also be increased when samples belong to inflamed or infected sites due to an influx of immune cells, tissue wounds, or necrosis^[12]^.

Accordingly, the amount of bacterial DNA present in some biological samples can reach very low levels, such as on skin samples due to the cutaneous low pH and the continuous secretion of antimicrobials^[13]^ or biopsies where the bacterial content is mainly associated with tissue or mucosa^[14]^. In this context, methods have been developed to enrich bacterial DNA, but they proved to be partially ineffective and compatible only with fresh specimens^[15,16]^. For this reason, the extraction of bacterial DNA from biological samples with high contamination of host eukaryotic DNA is usually much more demanding in terms of target sequencing depth and related costs to compensate for the lower fraction of microbial DNA. Thus, optimization of the extraction and sequencing protocols is now mandatory^[17]^.

Several approaches have been proposed to optimize DNA obtained from samples of different sources low in microbial counts. Many commercially available microbiome-specific DNA extraction kits^[18]^ and the use of a variety of additional steps employing detergents including saponin, Tween 20 and Triton X-100^[16]^, or Benzonase^[19]^ are amongst the approaches that have been developed to deplete host DNA without apparently influencing the prokaryotic DNA. Nevertheless, while many studies have demonstrated that these approaches could reduce human DNA contamination, few have pointed out their significant impact on the final microbial community structure delineated by shotgun metagenomics attempts. Among these approaches, different saponin percentages have been extensively tested and proposed as the golden standard for the depletion of eukaryotic DNA in highly contaminated samples with high relative concentrations of human DNA compared to bacterial DNA^[20-22]^. However, an important limitation of the use of saponin is linked to the differential impact that this reagent produces on the DNA of various bacteria and, thus, on the generation of artifacts in the relative abundance of the different microbial groups. In this regard, saponin has been previously reported to possess strong antimicrobial activity towards specific bacterial taxa through *in vitro* and *in vivo* investigations^[23]^.

For this reason, in this study, we have carefully evaluated the saponin-based protocol for extracting DNA from different human sample specimens to overcome high eukaryotic turnover and highlighted its main limitations in terms of microbial profiling.

## METHODS

### Human samples collection

Regarding the biological specimens included in this study, one sample was collected for each human matrix. Specifically, the vaginal swab sample was gathered by a healthy woman. The oral sample consisted of a lingual swab. Sputum samples represent the thick mucus expelled from the lower airways (bronchi and lungs) through a deep cough. Saliva samples were the early morning sampling before teeth washing. The skin sample represented the forehead microbiota sampled with a film dressing. Biopsies were different sections of the stomach (gastric antrum and body), and the nursing staff performed the nasopharyngeal swab collection at Parma Day Hospital. All the biological specimens included in this study were collected from different healthy donors and stored at -80 °C until they were processed.

Signed informed consent was obtained from the individuals enrolled in this study.

### Human DNA depletion and bacterial enrichment

Samples (1 mL) were centrifuged at 6,000 *g* for 3 min. For biopsies and skin samples, a fraction of the specimen was homogenized with 1 mL of phosphate buffer saline (PBS), and the suspension was then treated like other samples. After centrifugation, the supernatant was carefully removed, and cell pellets were incubated with PBS and saponin at the required concentration (see in the main text the different concentrations used) at room temperature for 10 min. The depletion protocol involved a commercially available product, known as saponin, obtained from the internal bark of the Quillaja Saponaria (Sigma-Aldrich, MO, USA).

Following the incubation, 350 µL of sterile water was added, and incubation was continued at room temperature for 30 s, after which 12 µL of 5 M NaCl was added to deliver an osmotic shock, lysing the damaged host cells. Samples were centrifuged at 6,000 *g* for 5 min, with the supernatant removed and the pellet resuspended in 100 µL of PBS. Turbo DNase buffered (Thermo Fisher Scientific, USA) with Turbo DNase enzyme (Thermo Fisher Scientific, USA) was added at 37 °C for 30 min to promote host cell lysis. Finally, the host DNA-depleted samples were washed two times with decreasing volumes of PBS (1 mL and 800 µL). After the final wash, the samples were centrifuged at 6,000 *g* for 3 min, the supernatant discarded, and the pellet resuspended in 600 µL of PBS. We included three cycles of bead-beating followed by three steps on ice. For the untreated counterpart of each biological sample, we avoided the whole depletion protocol, which means that after centrifugation, the bacterial pellet was incubated only with PBS (without the addition of saponin) and subsequently treated with sterile water and NaCl. We proceeded with centrifugations, washing, and incubations with PBS (without the addition of Turbo DNase), and finally bead-beating.

### DNA extraction and quantification

The DNA was extracted from the specimens using commercially available kits following the manufacturer’s instructions. The best-performing kits based on the biological origin matrix were employed for bacterial DNA extraction. QIAamp DNA Mini Kit (Qiagen, Hilden, Germany) was used to extract exclusively the bacterial DNA (avoiding other microbiome components such as fungi, archaea, and bacteriophages) from sputum, saliva, oral and nasopharyngeal swabs and biopsies, and ZymoBIOMICS DNA Miniprep Kit (Zymo Research, D4300) for vaginal swabs. Then, the DNA concentration and purity were investigated employing a Picodrop microtiter Spectrophotometer (Picodrop, Hinxton, UK).

### Quantitative real-time PCR for bacteria and human DNA

To evaluate the bacterial and eukaryotic abundance in samples treated or not with saponin and DNase, we performed a qPCR approach with specific primers for bacteria and humans, i.e., Probio_uni (5′-CCTACGGGRSGCAGCAG-3′) and Probio_rev (5′-ATTACCGCGGCTGCT-3′) for bacterial 16S rRNA^[24]^, bGLB+ (5′-ACACAACTGTGTTCACTAGC-3′) and bGLB- (5′-CAACTTCATCCACGTTCACC-3′) for the human β-globin^[25]^. DNA from the different biological samples was extracted and diluted to a concentration of 10 ng/µL. qPCR was performed using PowerUp SYBR Green Master Mix (ThermoFisher Scientific, US) on a CFX96 system (BioRad, CA, USA) following previously described protocols^[26]^. PCR products were detected with SYBR green fluorescent dye and amplified according to the following protocol: one cycle of 50 °C for 2 min, followed by one process of 95 °C for 2 min, followed by 40 cycles of 95 °C for 15 s, 55-60 °C for 15 s and 72 °C for 1 min. The melting curve was 65 to 95 °C with increments of 0.5 °C/s. In each run, negative controls (no DNA) were included. A standard curve was generated using the CFX96 software (BioRad), and chromosomal DNA belonging to untreated biological matrices and *Bifidobacterium longum* were used as qPCR standards for the eukaryotic and bacterial detection, respectively.

### Shallow shotgun sequencing

According to the manufacturer’s instructions, DNA library preparation was performed using the Nextera XT DNA sample preparation kit (Illumina, San Diego, CA, USA). First, one ng input DNA from each sample was used for the library preparation, which underwent fragmentation, adapter ligation, and amplification. Then, Illumina libraries were pooled equimolarly, denatured, and diluted to a concentration of 1.5 pM. Next, DNA sequencing was performed on a MiSeq instrument (Illumina) using a 2X 250 bp Output sequencing Kit and a deliberate spike-in of 1% PhiX control library.

### Short read taxonomic classification

Sequenced paired-end reads of each sample were subjected to a filtering step removing low-quality reads (minimum mean quality score 20, window size 5, quality threshold 25, and minimum length 100) using the fastq-mcf script (https://github.com/ExpressionAnalysis/ea-utils/blob/wiki/FastqMcf.md) to analyze high-quality sequenced data only. Then, employing the BWA aligner, an additional filtering step was performed to remove the reads mapping against the *Homo sapiens* genome sequence, thus removing possible contaminating human DNA, which was not removed by the previously described depletion protocol (see filtering tables reported below)^[27]^. Retained reads were taxonomically classified through the METAnnotatorX2 pipeline^[28]^ using a set of databases of reference genomes whose taxonomy was previously validated to maximize the accuracy of homology-based taxonomic classification of reads^[29]^. For those samples which, after the filtering step, reached a very low number of reads (e.g., gastric biopsies with ~200 reads), we verified that read depth was sufficient for comparisons. For this purpose, we considered the samples with the higher number of reads, such as sputum, saliva, and skin-treated samples (average of 191 high-quality reads). We reanalyzed them utilizing a subset of 200 reads, which returned comparable microbial profiles [Supplementary Table 1].

## RESULTS AND DISCUSSION

### Evaluation of bacterial and human abundance in biological samples with high content of eukaryotic DNA

To first estimate the effectiveness of the best approach for the bacterial DNA extraction aimed at reducing the carryover of human host DNA, we tested different amounts of saponin (2.5%, 1%, 0.1% and 0.0125% wt/vol) on a saliva sample collected from a healthy human donor. Saliva was chosen as an optimal test case due to the consistently high percentage of human DNA (~90%) determined by shotgun metagenomic sequencing^[8]^. Treatment with saponin aimed to induce lysis of eukaryotic cells, followed by adding DNase to the DNA extract to remove eukaryotic DNA before bacterial DNA extraction with a commercial kit supported by preliminary mechanical bead-beating lysis. Specifically, to assess the bacteria-human DNA ratio, qPCR was performed on the total DNA obtained using specific PCR primers targeting the bacterial 16S rRNA gene. In contrast, the human DNA level present in the saliva sample was determined by absolute quantification of the β-globulin gene^[25]^. In addition, this analysis was performed on DNA extracted from the same saliva sample that did not undergo the additional depletion process as a reference control to rule out if this saponin-based protocol can represent a potential cause of missed/additional bacterial detection. As a negative control, we used a sterile water sample treated with the same depletion protocol to ensure the extraction reagents did not contain contaminations. All qPCR analyses were performed on biological and technical triplicates.

**Figure 1:**
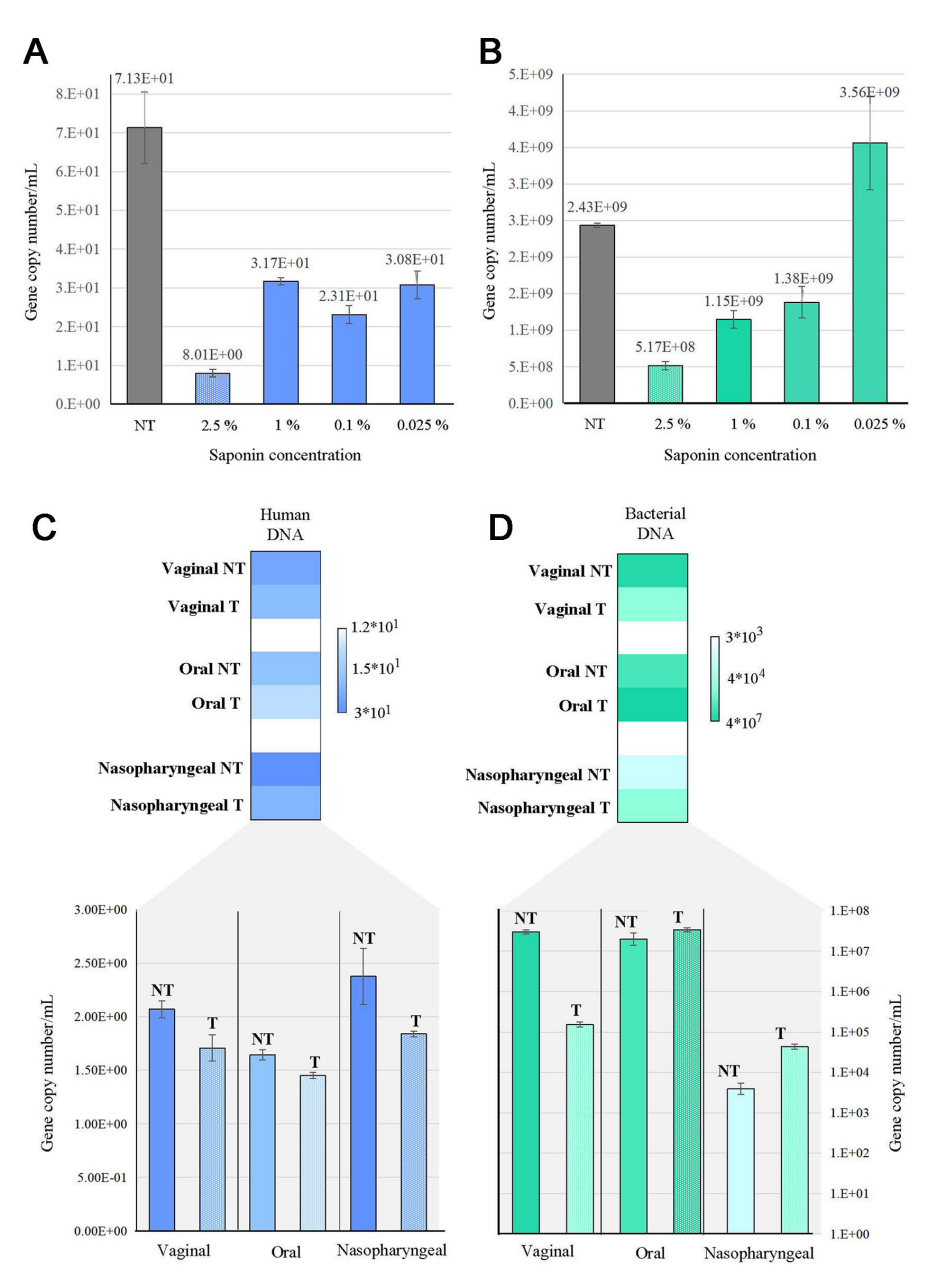
Abundance of bacterial and human DNA in different biological samples with high content of eukaryotic DNA evaluated through qPCR. Panel (A) shows the absolute quantification of host DNA (light blue); and panel (B) indicates the corresponding absolute quantification of bacterial DNA (green) in saliva samples treated with different amounts of saponin compared to the untreated counterpart (dark gray). Each pillar represents the average quantity of human and bacterial DNA content ± standard deviation; panel (C) displays the absolute quantification of host DNA (light blue) in a vaginal, oral and nasopharyngeal swab treated with 2.5% wt/vol concentration of saponin and subsequently treated with DNase before proceeding with microbial DNA extraction using the best performing commercial kit; panel (D) shows the bacterial DNA (green) counterpart in vaginal, oral, and nasopharyngeal samples. Every pillar represents a biological sample treated with saponin (T) or its untreated counterpart (NT). The color scales (blue and green) at the top of panels (C) and (D) indicate the increase in the amount of human or bacterial DNA in the analyzed samples.

Across all saponin amounts used, 2.5% wt/vol of detergent was found to successfully deplete most of the human DNA [Figure 1A]. Indeed, in a saliva sample treated with 2.5% wt/vol of saponin, the human DNA decreased to a concentration of 8.01 gene copy number/mL, while for the untreated sample, the host’s DNA recovered was around 71.3 gene copy number/mL [Figure 1A]. Conversely, host DNA abundance in the same saliva samples treated with 1%, 0.1%, and 0.025% wt/vol of saponin remained relatively stable at a concentration of 20-30 gene copy number/mL [Figure 1A]. Furthermore, the 2.5% wt/vol saponin-treated sample showed a 5.17 × 10^8^ gene copy number/mL bacterial content compared to the untreated saliva in which bacterial DNA had a concentration of 2.43 × 10^9^ gene copy number/mL [Figure 1B]. These findings showed that saponin lyses human cells by reducing the eukaryotic DNA content within the sample, but high amounts of such detergent (e.g., 2.5% wt/vol) also acted on bacterial cells, decreasing in part their abundance. In addition, by comparing the saliva sample treated with the lowest concentration of saponin (0.025% wt/vol) to its untreated counterpart, we noticed a reduction in bacterial DNA, probably due to the extraction protocol, but which does not appear to be statistically significant. Considering these preliminary results, detailed study-specific investigation of such a range of different concentrations of saponin on the retrieved DNA is mandatory. Thus, given the impact of saponin on the bacterial content, we suggest preliminarily testing the biological matrix according to the purpose of the study to assess the optimal quantity of saponin to be used. Specifically, treatment with saponin is risky if applied to biological samples originally low in bacterial content because the latter could be totally lost. On the other hand, this data showed that depletion protocol may be useful if applied to samples with a high content of bacteria to reduce the contamination of host-derived DNA.

### Investigation of how the saponin-based protocol impacts different biological matrices

Preliminary results were validated on other biological specimens from different human body sites rich in eukaryotic DNA, such as the vagina, oral cavity, and nasopharynx. Each sample was processed with a protocol reported by Charalampous *et al.* employing a 2.5% wt/vol concentration of saponin specifically for eukaryotic DNA removal^[20]^. Subsequently, the DNA extracts were treated with DNase to cleave the eukaryotic content released before proceeding with standard microbial DNA extraction. For the bacterial DNA extraction from different biological matrices, distinct commercially available kits were used, as described in the Experimental Procedures section. The amount of bacterial and eukaryotic DNA in the nucleic acid extracts was evaluated by qPCR using specific primers to discriminate bacterial 16S rRNA and β-globulin genes.

Data collected revealed that saponin treatment induced a depletion of eukaryotic DNA in all the samples analyzed [Figure 1C]. Notably, after the saponin treatment, host DNA content decreased by 17.5% in vaginal, 11.7% in oral, and 22.6% in nasopharyngeal swab samples with respect to the same untreated samples [Figure 1C]. In contrast, bacterial content seemed to slightly increase from 10^3^ to 10^4^ gene copy number/mL in the nasopharyngeal swab as well as in the oral and the nasopharyngeal samples after saponin-based depletion, suggesting that lower host’s DNA abundance may support the recovery of a higher amount of bacterial DNA in some specific biological matrices in contrast to what observed for saliva [Figure 1D]. However, confirming the previous findings, the saponin depletion protocol seemed to impact also on bacterial cells by promoting their lysis in biological samples such as vaginal swabs. In this case, probably due to the large amount of bacterial DNA, bacterial content decreased from 10^7^ to 10^5^ gene copy number/mL in the saponin-treated vaginal swab [Figure 1D].

Remarkably, the results confirmed what was observed for the saliva samples, i.e., saponin targets mainly human DNA, showing great performances in removing eukaryotic DNA from the biological samples while sometimes impacting the bacterial DNA content. The observed differences in bacterial population reduction can be considered biological matrix dependent. Thus, we suggest preliminary testing of each biological matrix included in a study to identify the best depletion approach in terms of saponin concentration according to the study purpose.

### Validation of the saponin DNA extraction protocol by shallow shotgun metagenomics of a broader range of biological matrices

The shotgun metagenomics approach allows to randomly sequence all the DNA fragments obtained from DNA extraction protocols, thus allowing the quantification of the prokaryotic/eukaryotic DNA ratio through specific bioinformatics applications^[29]^. To validate the outcome of our qPCR analyses, we performed shallow shotgun metagenomic sequencing^[30]^, a specific low-depth shotgun metagenomics technique that provides a reliable bacterial profile at the species level^[29]^. DNA samples from different biological matrices rich in eukaryotic DNA were submitted to DNA sequencing after being processed with the extraction protocol involving 2.5% wt/vol saponin and DNase, followed by bead-beating and kit extraction steps. To provide a more comprehensive overview of the human-associated microbiota, we included samples rich in eukaryotic DNA, such as a vaginal swab, saliva, skin, sputum, and nasopharyngeal swab samples [Table 1]. Our analysis also included a molecular biology-grade water sample undergoing the same depletion protocol. Notably, it did not provide enough sequencing data to be analyzed as it contained no eukaryotic or bacterial DNA contaminations.

**Table 1:** Filtering table of the analyzed DNA samples

**Biological samples**	**Sequenced reads produced**	**High-quality reads**	**Reads retained after *Homo sapiens* filtering**	**% Filtered *Homo sapiens***
Vaginal-NT	154,701	151,193	4,266	97.18%
Vaginal-T	222,202	211,864	159,017	24.94%
Gastric antrum-NT	101,522	99,529	494	99.50%
Gastric antrum-T	59,749	56,936	251	99.56%
Gastric body-NT	136,117	133,250	1,214	99.09%
Gastric body-T	143,220	139,737	199	99.86%
Saliva-NT	134,011	131,338	30,156	77.04%
Saliva-T	225,114	218,562	208,893	4.42%
Skin-NT	242,037	233,438	128,078	45.13%
Skin-T	236,997	227,914	163,985	28.05%
Sputum-NT	77,837	52,182	9,776	81.27%
Sputum-T	208,337	203,745	200,275	1.70%
Nasopharyngeal-Swab-NT	78,150	71,634	3,613	94.96%
Nasopharyngeal-Swab-T	4,184	4,017	3,933	2.09%

Different biological matrices rich in eukaryotic DNA were processed following the extraction protocol involving 2.5% wt/vol saponin and DNase followed by bead-beating and kit extraction steps. The DNA obtained was processed for shallow shotgun sequencing. NT: Untreated; T: treated.

Sequencing output resulted in an average of circa 144,585 pair-end reads per sample [Table 1], thus allowing an accurate assessment of the microbial profile associated with each specimen^[30]^. Each biological matrix treated according to the depletion approach was compared to its untreated counterpart to investigate the impact of the saponin method on the bacterial profile of each sample [Table 1].

The percentage of *Homo sapiens* reads obtained after the *in silico* filtering step in treated vaginal swab, saliva, skin, sputum, and nasopharyngeal swab samples ranged from 1% to a maximum of 28.05% [Table 1], with a reduction ranging from 45.1% to 97.2% compared to untreated samples. Thus, it was shown that the saponin-based protocol enabled a successful depletion of host DNA.

### Investigating the microbial profiles of the saponin-achieved DNA samples

Shallow shotgun metagenomic sequencing was also exploited to identify the microorganisms that populate biological samples at the species level and to validate their microbial taxonomic composition. Taxonomic profiles obtained through METAnnotatorX2 software demonstrated that skin, vaginal and nasopharyngeal swab samples processed with 2.5% (wt/vol) saponin retained the microbial profile corresponding to their untreated counterpart. To evaluate the divergence of treated and untreated profiles, we employed a taxonomic variation index (TVI), consisting of the absolute sum of positive and negative relative abundance differences observed for each microbial taxon, thus ranging from 0 for identical profiles to 200 for completely different taxonomic profiles. For the vaginal, skin, nasopharyngeal swab, and saliva samples, the retrieved TVI were 29.1, 5.05, 23.6, and 1.74, respectively [Supplementary Table 1]. Specifically, this taxonomic survey revealed that, amongst the most impacted taxa, *Bifidobacterium scardovii* was present in the untreated vaginal sample at a relative abundance of 49.8 %, compared to the 64.3 % of the treated counterpart [Supplementary Table 2 and Supplementary Figure 1]. In contrast, the relative abundance of *Bifidobacterium* spp., *Lactobacillus acidophilus*, and *Lactobacillus gasseri* in vaginal swabs mainly remained constant even after the depletion protocol [Supplementary Table 2 and Supplementary Figure 1]. Accordingly, *Cutibacterium* spp. in the skin sample and *Corynebacterium* spp. in nasopharyngeal swabs were present at the same relative abundance in both saponin-treated and saponin-untreated samples [Supplementary Table 2 and Supplementary Figure 1]. In these biological samples, only a few bacterial species (5 depending on specimen) present at low abundance (lower than 2%) in controls were absent in the saponin-treated samples [Supplementary Table 2]. This observation suggested that saponin treatment induces different effects based on the microbial species in the original biological sample.

However, the same limited impact on the bacterial DNA content was not observed for other biological matrices such as biopsies (gastric antrum and body), saliva, and sputum samples for which the same saponin-based extraction method induced a substantial alteration of the whole microbial taxonomic profile. In this case, the taxonomic variation index between treated samples and controls was 190.3 for the gastric antrum, 180.5 for the gastric body biopsies, and 129.7 for the sputum, respectively [Supplementary Table 2]. Consistently, 14 bacterial taxa with a prevalence between 0.5% to 25.7% were lost in the saponin-treated biopsy of the gastric antrum, 33 in the biopsy of the gastric body, 17 in the saliva, and 12 in the treated sputum [Supplementary Table 3 and Supplementary Figure 2]. Although with a relative abundance greater than 2%, the most represented bacterial taxa in the saponin-treated samples ranged from 4 bacterial taxa in the biopsies to 15 and 11 in the saliva and sputum, respectively [Supplementary Table 3 and Supplementary Figure 2]. In saliva, sputum, and biopsy specimens not treated with saponin, several members of *Haemophilus*, *Neisseria*, *Prevotella,* and *Veilonella* genera were present with a relative abundance higher than 10% and were the most representative bacterial taxa of these biological samples, while in the respective samples treated with saponin, they fell under the limit of detection [Supplementary Table 3 and Supplementary Figure 2].

In contrast, several bacterial taxa not detected in the untreated specimens appeared in the profile of saponin-treated samples, with few cases (such as *Streptococcus* spp. and *Actinomyces* spp.) where these taxa even became dominant in the retrieved taxonomic profiles [Supplementary Figure 2 and Supplementary Table 3]. Altogether, these data highlighted the impact of the saponin-based eukaryotic DNA depletion method, which can markedly alter the retained bacterial DNA profile.

In this context, we noticed that the depletion protocol less compromised samples with a substantially high bacterial load. Conversely, when processing samples poor in bacterial DNA, such as mucosa, saliva and sputum, the saponin treatment tended to reduce the already low bacterial population, resulting in the sequencing of a lower number of bacterial DNA sequences, causing the loss of the minor component of the bacterial population in the taxonomic profiles retrieved after data analysis. Therefore, these results significantly impact studies in which the purpose is represented by comparing and analyzing complex bacterial populations constituted by many microbial species at low relative abundance. For this reason, preliminary testing of biological matrices included in each study should be performed.

On the other hand, it should be considered that studies focused specifically on the detection of one or few target microbial taxa in simple microbial communities are less affected by the use of saponin depletion protocol, which may represent an extremely useful tool by increasing the microbial/host DNA ratio. For example, in the case of pathogen detection for clinical diagnosis, the use of saponin would be highly recommended to remove the large amount of human DNA present in the biological samples, in particular when applying shotgun or PCR/amplicon DNA sequencing approaches targeting and amplifying specific microbial taxa.

### Saponin/DNase treatment affects the Gram-positive/Gram-negative ratio of the retrieved metagenomic profiles

Microbial profiling revealed a differential response to saponin treatment of Gram-positive and Gram-negative bacteria in the biological samples analyzed. More specifically, although recent literature demonstrates a potential sensitivity of Gram-positive bacteria to saponin treatment, especially of microbes recovered from blood cultures^[31,32]^, in this study employing a range of different matrices, saponin seemed to have an impact on the relative abundance of Gram-negative bacteria that prompted a concomitant increase in the proportion of Gram-positive microbes with respect to the whole population. It has been generally accepted that bacteria differ in their susceptibility to cell lysis based on the molecular structures composing their superficial biological structures^[33]^. Compared to Gram-positives, Gram-negative bacteria possess a much thinner cell wall covered by an external membrane. This has been reported to increase susceptibility to specific cell lysis methods, thus explaining the drastic decrease in the number of Gram-negative associated DNA after saponin/DNase treatment^[31,32]^. Regarding this, it has been widely demonstrated that the antibacterial activity of saponins depends on their chemical structures; indeed, several natural compounds with surfactant properties extracted from plants are able to break down the bacterial cell wall, resulting in the leakage of the cell contents and affecting bacterial metabolism adversely^[34,35]^.

**Figure 2:**
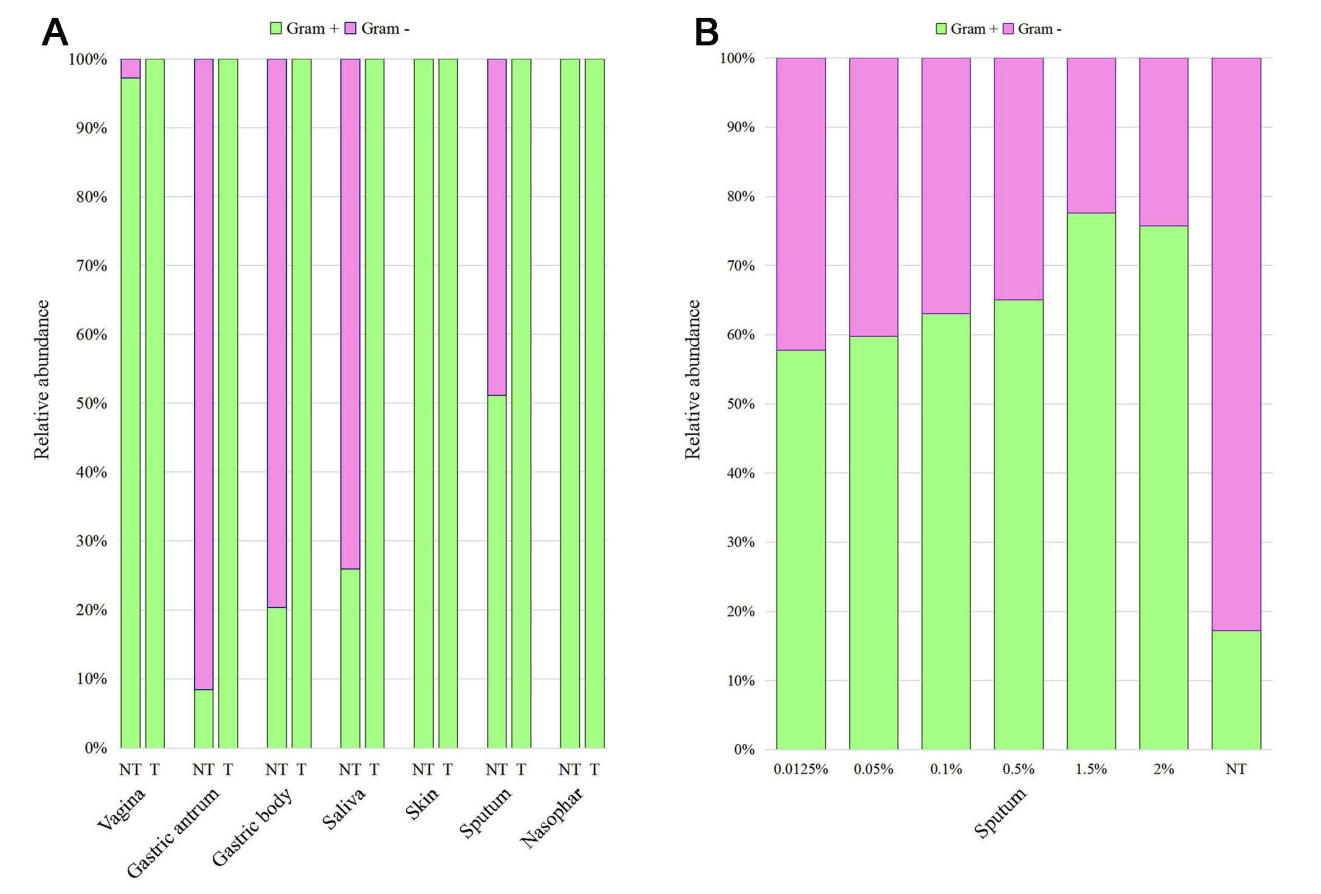
Gram-negative and Gram-positive bacterial ratio in different biological samples before and after saponin-based depletion protocol. Panel (A) indicates the percentages of Gram-negative (pink) and Gram-positive bacteria (green) on the total amount of bacterial DNA profiled in each biological sample before (NT) and after 2.5% wt/vol saponin-based depletion (T); panel (B) represents the percentages of Gram-negative (pink) and Gram-positive bacteria (green) detected in a sputum sample treated with different saponin amounts.

Supporting this hypothesis, we observed that the sum of the relative abundance of Gram-negative bacteria in the antrum and the gastric body biopsies was completely depleted after saponin treatment from 91.6% and 79.7%, respectively, to 0%. Furthermore, in saliva and sputum samples where Gram-negative bacteria represented 74.1% and 48.8%, respectively, of the total amount of bacterial content in the control samples, their presence was completely depleted after treatment with saponin due to a dominance of Gram-positive bacteria [Figure 2A and Supplementary Table 4]. In contrast, the biological samples where the microbial populations were mainly represented by Gram-negative bacteria, such as skin and nasopharyngeal swabs, were least affected due to a reduced shift in the Gram-positive/Gram-negative ratio, thus explaining the limited impact of saponin treatment on the taxonomic profiles obtained for these biological matrices [Figure 2A and Supplementary Table 4].

Overall, these data underline that saponin can markedly lower the Gram-positive/Gram-negative ratio in the recovered bacterial DNA, thus altering the post-sequencing microbial profiles. In this regard, a preliminary taxonomic investigation of the microbial population is important to evaluate whether saponin will be acceptable in altering the taxonomic profiles. In fact, in the case of samples with a bacterial population dominated by Gram-positive taxa, alterations by saponin treatment will be more contained and acceptable in terms of the accuracy of data analysis.

### Comparison of the effect of different saponin percentages on the microbial profile

Although 2.5% wt/vol is the generally used amount of saponin in most protocols used for eukaryotic DNA removal, we decided to evaluate whether a lower amount of this detergent could avoid the bias in the estimation of Gram-negative/Gram-positive bacteria ratio in the biological samples assayed. To do this, DNA isolated from the same sputum sample was processed with variable concentrations of saponin, i.e., 0.0125%, 0.05%, 0.1%, 0.5%, 1.5% and 2% wt/vol. DNA obtained from microbial DNA extraction was subjected to shallow shotgun metagenomic analysis.

Notably, the analysis of the achieved metagenomic data revealed that saponin had an impact on both human DNA abundance and Gram-positive/Gram-negative ratio directly proportional to the amount used. In fact, host DNA content ranged from 36.4% in the 0.0125% to 3.2% in the 2% wt/vol saponin-treated saliva, compared to the control sample in which the host DNA content was 53.2% [Table 2].

**Table 2:** Filtering table of the analyzed sputum samples processed with the extraction protocol involving different saponin amounts, DNase, and bead-beating treatments to test the effect of lower detergent concentrations on estimating the bias in the Gram-negative/Gram-positive bacteria ratio

**Sample**	**Sequenced reads produced**	**High-quality reads**	**Reads retained after *Homo sapiens* filtering**	**% filtered *Homo***
Sputum-0.0125%	51,453	48,877	31,094	36.38%
Sputum-0.05%	34,712	33,795	31,860	5.73%
Sputum-0.1%	41,873	40,807	39,754	2.58%
Sputum-0.5%	13,420	13,056	12,774	2.16%
Sputum-1.5%	34,688	33,633	32,716	2.73%
Sputum-2%	37,528	36,418	35,249	3.21%
Sputum-NT	38,545	37,556	17,595	53.15%

NT: Untreated.

To better detail the impact of saponin concentration, we focused our interest on the key Gram-negative bacterial taxa previously identified as dominant in the untreated sputum sample. The Gram-negative *Prevotella histicola* was present in the control sample with a relative abundance of 19% and decreased from 9.1% to 5%, respectively, in the sample treated with 0.0125% wt/vol and 2% wt/vol of saponin [Supplementary Table 5 and Supplementary Figure 3]. Similarly, the Gram-negative *Veillonella atypica* decreased from an initial relative abundance of 8.1% in the untreated to 4.5% and 2.6%, respectively, in the 0.0125% and 2% wt/vol treated samples [Supplementary Table 5 and Supplementary Figure 3]. In contrast, the Gram-positive *Streptococcus salivarius* was detected as a dominant bacterial taxon in the sputum treated with 2% wt/vol of saponin, but its relative abundance progressively decreased from 8.8% in the DNA extracted with 0.0125% wt/vol of saponin to 1.8% in the control sample, thus suggesting that its increase in relative abundance after saponin treatment was related to a reduction of the Gram-negative population [Supplementary Table 5 and Supplementary Figure 3]. Similarly, the Gram-positive *Schalia* sp. was present in the 2% wt/vol treated saponin sample with a relative abundance of 8% and decreased to 5.2% due to 0.05% wt/vol saponin treatment while an abundance of 2.4% was observed in the control. Moreover, several Gram-negative taxa, such as other members of the *Prevotella* and *Veillonella* genera, as well as unknown species of the *Lancefieldella* and *Schalia* genera, decreased with the increasing amount of saponin, while the Gram-positive *Actinomyces* and *Streptococcus* spp. increased in relative abundance, reinforcing the previous findings regarding the depletion protocol’s impact on the taxonomic profile [Supplementary Table 5 and Supplementary Figure 3].

These results confirmed that saponin treatment has an impact on the Gram-positive/Gram-negative ratio. Gram-negative percentages decreased with the increasing concentration of saponin applied, thus inducing a concomitant increase in the relative abundance of Gram-positive bacteria in the overall taxonomic profile. Indeed, Gram-negatives ranged from an average of 42.3% in the 0.0125% wt/vol saponin-treated samples to an average of 35% and 24.3% in the 0.5% and 2% wt/vol saponin treated samples, respectively, compared to the starting 82.3% in the untreated saliva sample [Supplementary Table 6 and Figure 2B].

Remarkably, despite the fact that even low saponin amounts are able to modulate the retrieved bacterial DNA in terms of Gram-negative taxonomic representation, saponin could be efficiently employed for the depletion of human DNA when targeting the detection of microbial populations dominated by Gram-positive bacteria or when targeting specific microbial taxa such as pathogens in clinical contexts. Thus, data retrieved in this study indicate the need for preliminary tests on the biological matrices under investigation to assess the feasibility and effects of the saponin protocol use. In this context, in the case of studies employing long-read sequencing, a further novel approach that can be employed to overcome contaminants derived from high amounts of host DNA, as an alternative to saponin, could be represented by the SQK-RPB004 kit combined with the SQK-LSK109 kit proposed by Oxford Nanopore Technologies. This consists of the specific enrichment or depletion of target DNA directly during sequencing by simply providing reference fasta sequence^[36]^. Nevertheless, it should be considered that this approach may considerably impact the amount of sequencing data retrieved.

In conclusion, in the framework of this study, we investigated the performances of the saponin-based eukaryotic DNA depletion approach on different biological matrices and the impact on the correlated microbial taxonomic profiles retrieved after DNA sequencing and data analysis.

Overall, this depletion method successfully reduced the amount of human DNA but drastically changed the detected microbial composition harbored by the specimens analyzed, inducing a drastic reduction of Gram-negative bacteria DNA.

However, although the saponin-based approach is one of the most used methods, other protocols pursue the same aim^[18,19,37]^. In these cases, it will also be necessary to carry out a very careful evaluation of protocol limitations to avoid alteration of the retrieved microbial DNA profile. Our findings highlight a serious issue that might affect the reliability of the microbial profiles and the biological meaning of the associated metagenomic studies that use these specific DNA extraction protocols involving the use of saponin.

Based on the results obtained in our study, we highlighted that the use of saponin should be avoided if specific evaluations of the microbial/host DNA ratio and complexity of the targeted microbial population are not known. For this reason, we suggest that for each study intending to apply saponin treatment, it is necessary to introduce detailed preliminary tests on the biological matrices investigated to assess the feasibility and efficacy of the saponin-based host DNA depletion approach.

## DECLARATIONS

### Availability of data and materials

Shotgun metagenomics data are accessible through SRA study accession number PRJNA864589.

### Copyright

© The Author(s) 2023.
