# Supplementary material for: Saponin treatment for eukaryotic DNA depletion alters the microbial DNA profiles by reducing the abundance of Gram-negative bacteria in metagenomics analyses
Source: Microbiome Res Rep. 2023 Nov 15;3(1):4. doi: 10.20517/mrr.2023.02 (PMC10917613; doi:10.20517/mrr.2023.02)
Supplement: Supplementary file 2 [file mrr-3-1-4-SupplementaryMaterials.pdf]

Supplementary Figures

Saponin treatment for eukaryotic DNA depletion alters the microbial DNA profiles by reducing the abundance of Gram-negative bacteria in metagenomics analyses

Giulia Longhi[1,2], Chiara Argentini[1], Federico Fontana[1,2], Chiara Tarracchini[1], Leonardo Mancabelli[3,4], Gabriele Andrea Lugli[1], Giulia Alessandri[1], Edith Lahner[5], Giulia Pivetta[5], Francesca Turroni[1,4], Marco Ventura[1,4], Christian Milani[1,4]

[1]Laboratory of Probiogenomics, Department of Chemistry, Life Sciences, and Environmental Sustainability, University of Parma, Parma 43124, Italy.
[2]GenProbio Srl, Parma 43124, Italy.
[3]Department of Medicine and Surgery, University of Parma, Parma 43124, Italy.
[4]Microbiome Research Hub, University of Parma, Parma 43124, Italy.
[5]Medical-Surgical Department of Clinical Sciences and Translational Medicine, Sant'Andrea Hospital, School of Medicine, University Sapienza, Rome 00185, Italy.

Correspondence to: Prof. Christian Milani, Prof. Marco Ventura, Laboratory of Probiogenomics, Department of Chemistry, Life Sciences, and Environmental Sustainability, University of Parma, Parco Area delle Scienze 11a, Parma 43124, Italy.
E-mail: christian.milani@unipr.it; marco.ventura@unipr.it

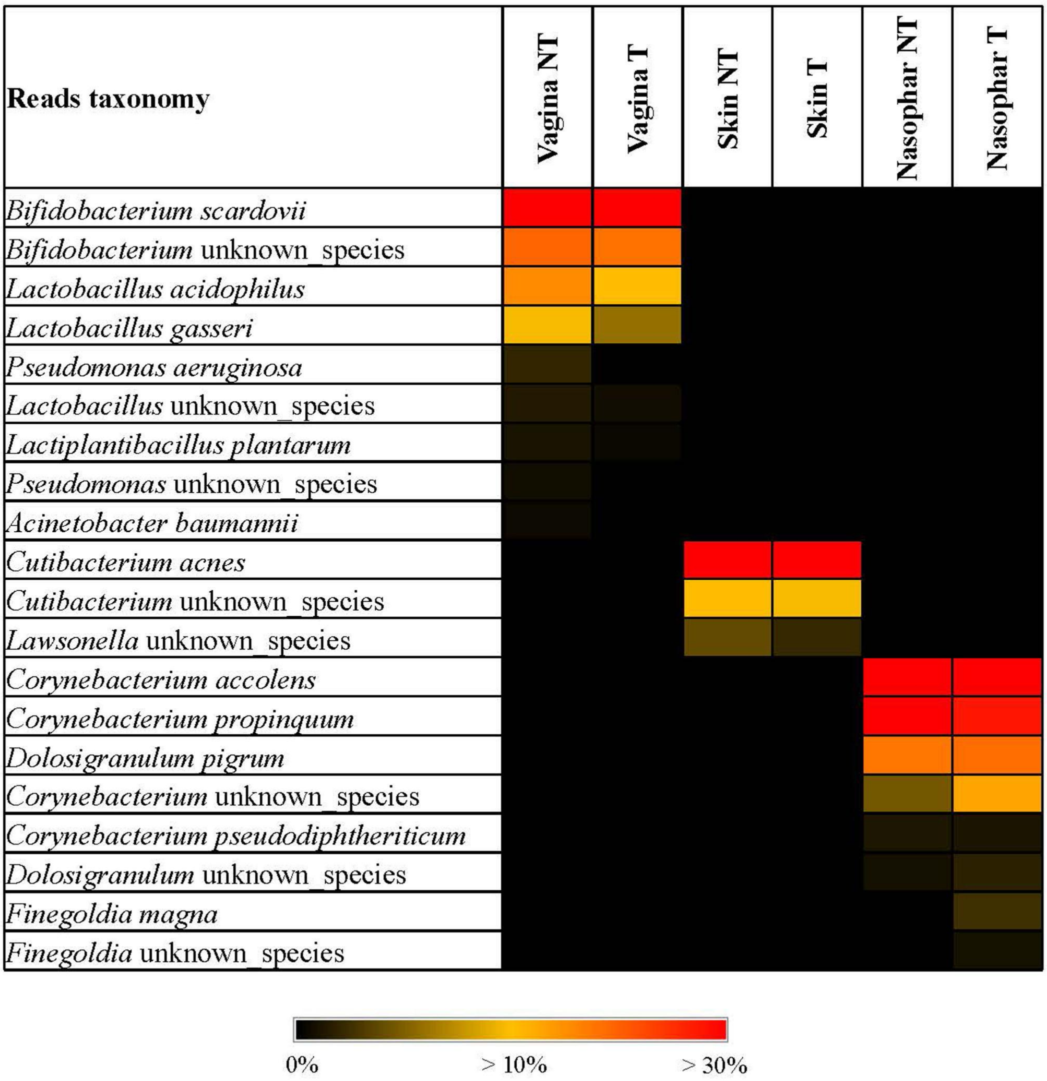

Supplementary Figure 1. Taxonomic microbial profiling of biological samples showing limited biases in microbial taxonomy after 2.5% wt/vol saponin treatment. The histogram displays the relative abundance of each microbial species identified in the vagina, skin, and nasopharynx samples. Both saponin-treated (T) and saponin-untreated samples (NT) are reported for each matrix. From left to right, the color-coding order of the legend reflects average abundances from largest to smallest.

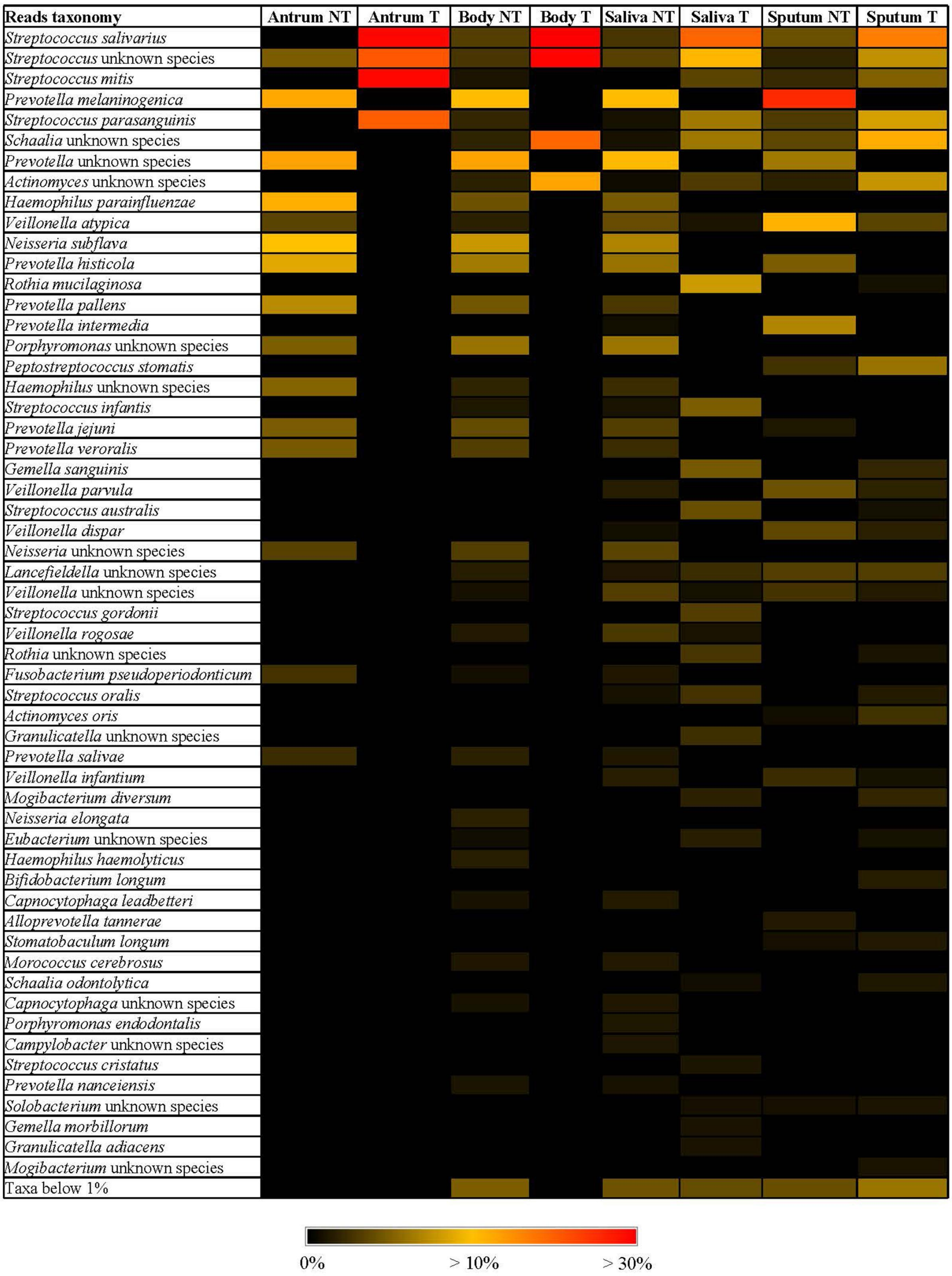

Supplementary Figure 2. Taxonomic microbial profiling of biological samples showing marked biases in microbial taxonomy after 2.5% wt/vol saponin treatment. The histogram displays the relative abundance of each microbial species identified in the biopsies, gastric antrum and body, saliva, and sputum. Both saponin-treated (T) and saponin-untreated samples (NT) are reported for each matrix. From left to right, the color-coding order of the legend reflects average abundances from largest to smallest. Most representative bacterial species are indicated with different colors.

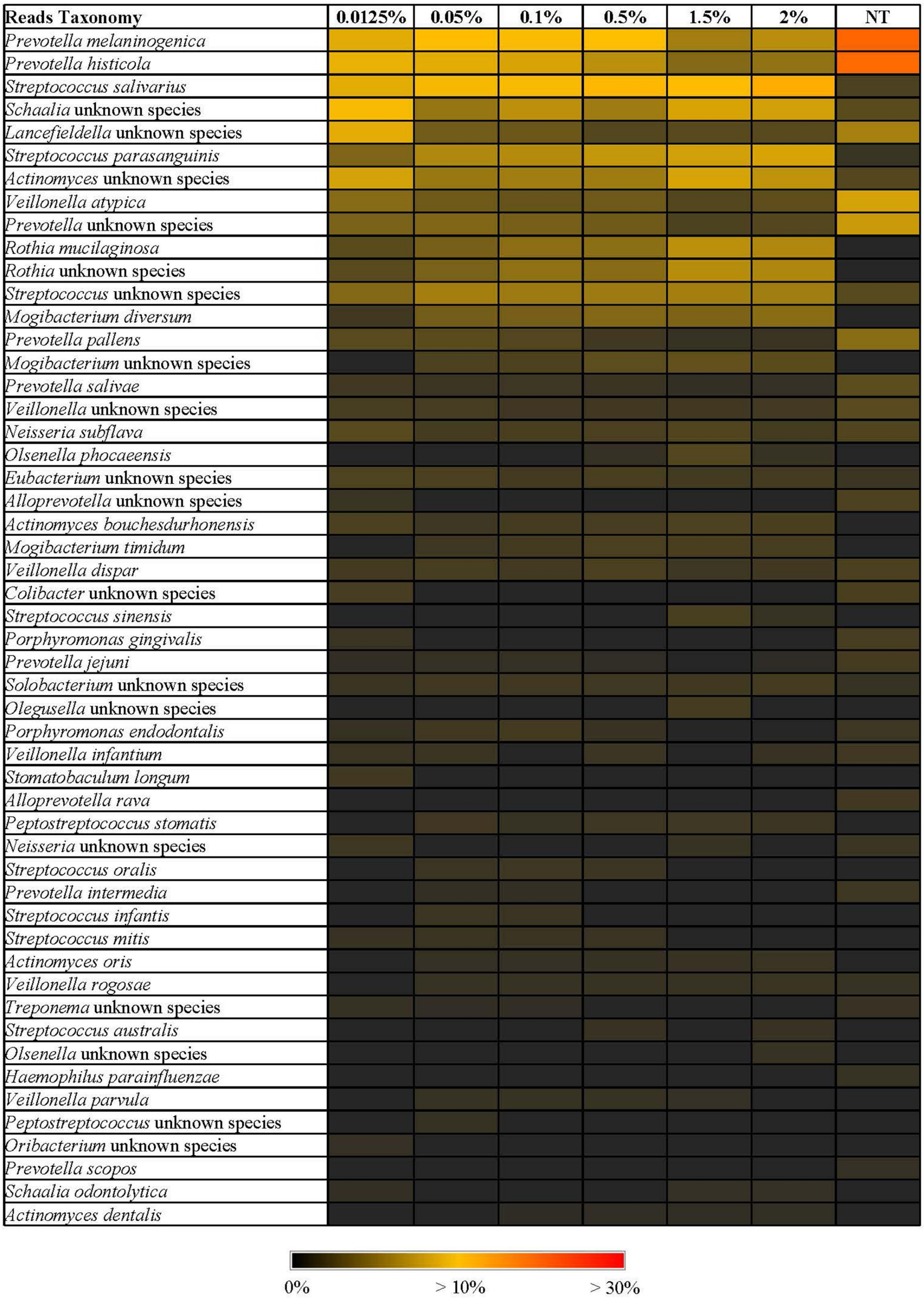

Supplementary Figure 3. Taxonomic microbial profiling of the same sputum sample treated with different saponin amounts. From left to right, the color-coding order of the legend reflects average abundances from largest to smallest. Most representative bacterial species are highlighted with different colors. The untreated sample is reported as NT.
